# 2,2-Dichloro-*N*-(3,4-dimethyl­phen­yl)acetamide

**DOI:** 10.1107/S1600536809022557

**Published:** 2009-06-17

**Authors:** B. Thimme Gowda, Sabine Foro, Ingrid Svoboda, Hartmut Fuess

**Affiliations:** a Department of Chemistry, Mangalore University, Mangalagangotri 574 199, Mangalore, India; b Institute of Materials Science, Darmstadt University of Technology, Petersenstrasse 23, D-64287 Darmstadt, Germany

## Abstract

In the title compound, C_10_H_11_Cl_2_NO, the N—H bond is *syn* to the 3-methyl substituent in the aromatic ring, similar to that observed in *N*-(3,4-dimethyl­phen­yl)acetamide and to the 3-chloro substituent in 2,2-dichloro-*N*-(3,4-dichloro­phen­yl)acetamide, and contrasting with the *anti* conformation observed for the 3-methyl substituent in 2,2,2-trichloro-*N*-(3,4-dimethyl­phen­yl)acetamide. On the other hand, it is *anti* to the C=O bond. An inter­molecular N—H⋯O hydrogen bond links mol­ecules into infinite chains along the *b* axis.

## Related literature

For the preparation of the compound, see: Shilpa & Gowda (2007 ▶). For related structures, see: Gowda *et al.* (2007 ▶, 2008 ▶, 2009 ▶)
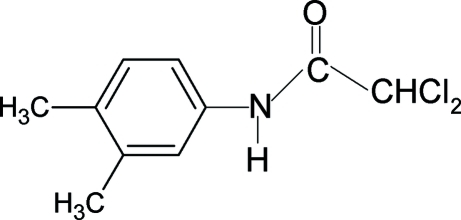


## Experimental

### 

#### Crystal data

C_10_H_11_Cl_2_NO
*M* *_r_* = 232.10
Monoclinic, 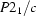

*a* = 11.951 (1) Å
*b* = 10.534 (1) Å
*c* = 9.303 (1) Å
β = 111.26 (1)°
*V* = 1091.5 (2) Å^3^
*Z* = 4
Mo *K*α radiation
μ = 0.56 mm^−1^
*T* = 299 K
0.28 × 0.20 × 0.12 mm

#### Data collection

Oxford Diffraction Xcalibur diffractometer with a Sapphire CCD detector
Absorption correction: multi-scan (*CrysAlis RED*; Oxford Diffraction, 2007 ▶) *T*_min_ = 0.859, *T*_max_ = 0.936
4567 measured reflections
2214 independent reflections
1495 reflections with *I* > 2σ(*I*)
*R*_int_ = 0.020

#### Refinement

*R*[*F* ^2^ > 2σ(*F* ^2^)] = 0.056
*wR*(*F* ^2^) = 0.187
*S* = 1.20
2214 reflections
144 parameters
H atoms treated by a mixture of independent and constrained refinement
Δρ_max_ = 0.32 e Å^−3^
Δρ_min_ = −0.37 e Å^−3^

### 

Data collection: *CrysAlis CCD* (Oxford Diffraction, 2004 ▶); cell refinement: *CrysAlis RED* (Oxford Diffraction, 2007 ▶); data reduction: *CrysAlis RED*; program(s) used to solve structure: *SHELXS97* (Sheldrick, 2008 ▶); program(s) used to refine structure: *SHELXL97* (Sheldrick, 2008 ▶); molecular graphics: *PLATON* (Spek, 2009 ▶); software used to prepare material for publication: *SHELXL97*.

## Supplementary Material

Crystal structure: contains datablocks I, global. DOI: 10.1107/S1600536809022557/bg2269sup1.cif

Structure factors: contains datablocks I. DOI: 10.1107/S1600536809022557/bg2269Isup2.hkl

Additional supplementary materials:  crystallographic information; 3D view; checkCIF report

## Figures and Tables

**Table 1:** Hydrogen-bond geometry (Å, °)

*D*—H⋯*A*	*D*—H	H⋯*A*	*D*⋯*A*	*D*—H⋯*A*
N1—H1*N*⋯O1^i^	0.84 (4)	2.07 (4)	2.894 (3)	166 (3)

Symmetry code: (i) 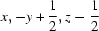.

**Fig. 1.**
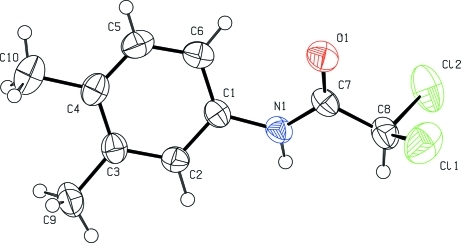
Molecular structure of (I), showing the atom labelling scheme. The displacement ellipsoids are drawn at the 50% probability level.

**Fig. 2.**
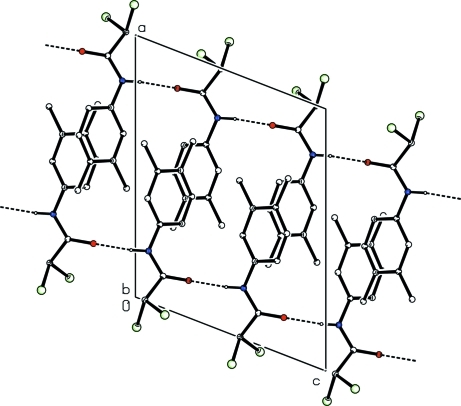
Molecular packing of (I) with hydrogen bonding shown as dashed lines.

## supplementary crystallographic information

### Comment

As part of a study of the effect of ring and side chain substitutions on the crystal structures of aromatic amides (Gowda *et al.*, 2007; 2008; 2009), the structure of 2,2-dichloro-*N*-(3,4-dimethylphenyl)acetamide (I) has been determined. The conformation of the N—H bond in the title compound is *syn* to the 3-methyl substituent in the aromatic ring [ similar to that observed in *N*-(3,4-dimethylphenyl)acetamide (Gowda *et al.*, 2008) and to the 3-chloro substituent in 2,2-dichloro-*N*- (3,4-dichlorophenyl)-acetamide (Gowda *et al.*, 2007)], and contrasting the *anti* conformation observed for the 3-methyl substituent in 2,2,2-trichloro-*N*-(3,4-dimethylphenyl)acetamide (Gowda *et al.*, 2009). On the other hand, it is anti to the C=O bond, as observed in other amides. A N—H···O intermolecular hydrogen bond links molecules into infinite chains along the *b* axis. (Table 1, Fig. 2).

### Experimental

Compound (I) was prepared and characterized according to the literature method (Shilpa and Gowda, 2007). Single crystals were obtained from the slow evaporation of an ethanolic solution of (I).

### Refinement

The H atoms of the methyl groups were positioned with idealized geometry using a riding model [C—H = 0.96 Å]. The other H atoms were located in difference map and their positional parameters were refined freely [N—H = 0.84 (4) Å and C—H = 0.87 (4)–0.97 (4) Å]. All H atoms were refined with isotropic displacement parameters (set to 1.2 times of the *U*_eq_ of the parent atom).

### Crystal data

**Table:**

C_10_H_11_Cl_2_NO	*F*(000) = 480
*M**_r_* = 232.10	*D*_x_ = 1.412 Mg m^−^^3^
Monoclinic, *P*2_1_/*c*	Mo *K*α radiation, λ = 0.71073 Å
Hall symbol: -P 2ybc	Cell parameters from 1463 reflections
*a* = 11.951 (1) Å	θ = 2.6–27.9°
*b* = 10.534 (1) Å	µ = 0.56 mm^−^^1^
*c* = 9.303 (1) Å	*T* = 299 K
β = 111.26 (1)°	Prism, colourless
*V* = 1091.5 (2) Å^3^	0.28 × 0.20 × 0.12 mm
*Z* = 4	

### Data collection

**Table:**

Oxford Diffraction Xcalibur diffractometer with a Sapphire CCD detector	2214 independent reflections
Radiation source: fine-focus sealed tube	1495 reflections with *I* > 2σ(*I*)
graphite	*R*_int_ = 0.020
Rotation method data acquisition using ω and φ scans	θ_max_ = 26.4°, θ_min_ = 2.7°
Absorption correction: multi-scan (*CrysAlis RED*; Oxford Diffraction, 2007)	*h* = −14→13
*T*_min_ = 0.859, *T*_max_ = 0.936	*k* = −13→6
4567 measured reflections	*l* = −11→11

### Refinement

**Table:**

Refinement on *F*^2^	Primary atom site location: structure-invariant direct methods
Least-squares matrix: full	Secondary atom site location: difference Fourier map
*R*[*F*^2^ > 2σ(*F*^2^)] = 0.056	Hydrogen site location: inferred from neighbouring sites
*wR*(*F*^2^) = 0.187	H atoms treated by a mixture of independent and constrained refinement
*S* = 1.20	*w* = 1/[σ^2^(*F*_o_^2^) + (0.1*P*)^2^] where *P* = (*F*_o_^2^ + 2*F*_c_^2^)/3
2214 reflections	(Δ/σ)_max_ = 0.023
144 parameters	Δρ_max_ = 0.32 e Å^−^^3^
0 restraints	Δρ_min_ = −0.37 e Å^−^^3^

### Special details

**Table:**

Experimental. CrysAlis RED (Oxford Diffraction, 2007) Empirical absorption correction using spherical harmonics, implemented in SCALE3 ABSPACK scaling algorithm.
Geometry. All e.s.d.'s (except the e.s.d. in the dihedral angle between two l.s. planes) are estimated using the full covariance matrix. The cell e.s.d.'s are taken into account individually in the estimation of e.s.d.'s in distances, angles and torsion angles; correlations between e.s.d.'s in cell parameters are only used when they are defined by crystal symmetry. An approximate (isotropic) treatment of cell e.s.d.'s is used for estimating e.s.d.'s involving l.s. planes.
Refinement. Refinement of *F*^2^ against ALL reflections. The weighted *R*-factor *wR* and goodness of fit *S* are based on *F*^2^, conventional *R*-factors *R* are based on *F*, with *F* set to zero for negative *F*^2^. The threshold expression of *F*^2^ > σ(*F*^2^) is used only for calculating *R*-factors(gt) *etc*. and is not relevant to the choice of reflections for refinement. *R*-factors based on *F*^2^ are statistically about twice as large as those based on *F*, and *R*- factors based on ALL data will be even larger.

### Fractional atomic coordinates and isotropic or equivalent isotropic displacement parameters (Å^2^)

**Table:**

	*x*	*y*	*z*	*U*_iso_*/*U*_eq_	
Cl1	−0.02466 (12)	0.39913 (11)	0.15433 (13)	0.0798 (4)	
Cl2	−0.11319 (10)	0.15743 (13)	0.00649 (16)	0.0885 (5)	
O1	0.1417 (2)	0.1762 (2)	0.2797 (2)	0.0521 (7)	
N1	0.1947 (2)	0.1901 (3)	0.0686 (3)	0.0389 (6)	
H1N	0.173 (3)	0.217 (3)	−0.023 (4)	0.047*	
C1	0.3132 (3)	0.1393 (3)	0.1286 (3)	0.0360 (7)	
C2	0.3945 (3)	0.1829 (3)	0.0644 (3)	0.0383 (7)	
H2	0.370 (3)	0.240 (3)	−0.020 (4)	0.046*	
C3	0.5126 (3)	0.1407 (3)	0.1172 (4)	0.0400 (7)	
C4	0.5503 (3)	0.0526 (3)	0.2372 (4)	0.0442 (8)	
C5	0.4666 (3)	0.0078 (3)	0.2959 (4)	0.0493 (9)	
H5	0.487 (3)	−0.050 (4)	0.367 (4)	0.059*	
C6	0.3489 (3)	0.0492 (3)	0.2448 (4)	0.0439 (8)	
H6	0.295 (3)	0.013 (3)	0.284 (4)	0.053*	
C7	0.1210 (3)	0.2076 (3)	0.1455 (3)	0.0384 (7)	
C8	0.0029 (3)	0.2709 (4)	0.0500 (4)	0.0481 (8)	
H8	−0.003 (3)	0.302 (4)	−0.051 (4)	0.058*	
C9	0.5985 (3)	0.1928 (4)	0.0464 (5)	0.0580 (10)	
H9A	0.6372	0.1238	0.0153	0.070*	
H9B	0.6580	0.2442	0.1209	0.070*	
H9C	0.5550	0.2435	−0.0420	0.070*	
C10	0.6786 (3)	0.0066 (4)	0.3023 (5)	0.0664 (11)	
H10A	0.7313	0.0774	0.3425	0.080*	
H10B	0.6986	−0.0336	0.2221	0.080*	
H10C	0.6876	−0.0532	0.3835	0.080*	

### Atomic displacement parameters (Å^2^)

**Table:**

	*U*^11^	*U*^22^	*U*^33^	*U*^12^	*U*^13^	*U*^23^
Cl1	0.0971 (9)	0.0780 (8)	0.0699 (7)	0.0313 (6)	0.0370 (6)	−0.0031 (5)
Cl2	0.0435 (6)	0.1112 (10)	0.1088 (10)	−0.0225 (6)	0.0251 (6)	−0.0137 (8)
O1	0.0568 (15)	0.0711 (16)	0.0349 (12)	0.0059 (12)	0.0246 (11)	0.0034 (11)
N1	0.0367 (14)	0.0520 (16)	0.0303 (13)	−0.0016 (12)	0.0147 (11)	0.0027 (12)
C1	0.0375 (16)	0.0395 (16)	0.0341 (15)	−0.0027 (13)	0.0167 (13)	−0.0061 (13)
C2	0.0411 (18)	0.0404 (17)	0.0348 (15)	0.0007 (13)	0.0154 (14)	0.0031 (13)
C3	0.0377 (17)	0.0404 (17)	0.0442 (17)	0.0008 (13)	0.0174 (15)	−0.0070 (14)
C4	0.0420 (18)	0.0405 (17)	0.0447 (17)	0.0050 (14)	0.0092 (15)	−0.0068 (15)
C5	0.061 (2)	0.0444 (19)	0.0425 (18)	0.0087 (17)	0.0188 (17)	0.0064 (16)
C6	0.051 (2)	0.0427 (18)	0.0412 (17)	−0.0029 (15)	0.0208 (15)	0.0018 (15)
C7	0.0385 (17)	0.0451 (17)	0.0352 (16)	−0.0079 (14)	0.0176 (13)	−0.0056 (14)
C8	0.0407 (18)	0.065 (2)	0.0426 (18)	−0.0003 (16)	0.0199 (15)	−0.0017 (17)
C9	0.044 (2)	0.059 (2)	0.082 (3)	0.0034 (16)	0.036 (2)	0.0025 (19)
C10	0.052 (2)	0.068 (3)	0.069 (2)	0.0146 (19)	0.011 (2)	0.001 (2)

### Geometric parameters (Å, °)

**Table:**

Cl1—C8	1.763 (4)	C4—C10	1.510 (5)
Cl2—C8	1.763 (4)	C5—C6	1.382 (5)
O1—C7	1.227 (4)	C5—H5	0.87 (4)
N1—C7	1.334 (4)	C6—H6	0.93 (4)
N1—C1	1.425 (4)	C7—C8	1.522 (5)
N1—H1N	0.84 (4)	C8—H8	0.97 (4)
C1—C6	1.384 (4)	C9—H9A	0.9600
C1—C2	1.390 (4)	C9—H9B	0.9600
C2—C3	1.389 (4)	C9—H9C	0.9600
C2—H2	0.95 (3)	C10—H10A	0.9600
C3—C4	1.395 (5)	C10—H10B	0.9600
C3—C9	1.510 (5)	C10—H10C	0.9600
C4—C5	1.385 (5)		
			
C7—N1—C1	126.7 (3)	O1—C7—N1	125.4 (3)
C7—N1—H1N	118 (2)	O1—C7—C8	121.0 (3)
C1—N1—H1N	115 (2)	N1—C7—C8	113.6 (3)
C6—C1—C2	119.8 (3)	C7—C8—Cl1	109.5 (2)
C6—C1—N1	123.0 (3)	C7—C8—Cl2	108.9 (3)
C2—C1—N1	117.2 (3)	Cl1—C8—Cl2	110.92 (18)
C3—C2—C1	121.4 (3)	C7—C8—H8	116 (2)
C3—C2—H2	118 (2)	Cl1—C8—H8	108 (2)
C1—C2—H2	121 (2)	Cl2—C8—H8	103 (2)
C2—C3—C4	119.2 (3)	C3—C9—H9A	109.5
C2—C3—C9	119.6 (3)	C3—C9—H9B	109.5
C4—C3—C9	121.2 (3)	H9A—C9—H9B	109.5
C5—C4—C3	118.2 (3)	C3—C9—H9C	109.5
C5—C4—C10	120.4 (3)	H9A—C9—H9C	109.5
C3—C4—C10	121.4 (3)	H9B—C9—H9C	109.5
C6—C5—C4	123.2 (3)	C4—C10—H10A	109.5
C6—C5—H5	117 (3)	C4—C10—H10B	109.5
C4—C5—H5	119 (3)	H10A—C10—H10B	109.5
C5—C6—C1	118.2 (3)	C4—C10—H10C	109.5
C5—C6—H6	120 (2)	H10A—C10—H10C	109.5
C1—C6—H6	122 (2)	H10B—C10—H10C	109.5
			
C7—N1—C1—C6	31.6 (5)	C10—C4—C5—C6	177.8 (3)
C7—N1—C1—C2	−149.1 (3)	C4—C5—C6—C1	0.4 (5)
C6—C1—C2—C3	−1.8 (5)	C2—C1—C6—C5	1.6 (5)
N1—C1—C2—C3	178.8 (3)	N1—C1—C6—C5	−179.1 (3)
C1—C2—C3—C4	0.0 (5)	C1—N1—C7—O1	−4.0 (5)
C1—C2—C3—C9	−178.6 (3)	C1—N1—C7—C8	176.7 (3)
C2—C3—C4—C5	2.0 (5)	O1—C7—C8—Cl1	50.4 (4)
C9—C3—C4—C5	−179.4 (3)	N1—C7—C8—Cl1	−130.4 (3)
C2—C3—C4—C10	−178.1 (3)	O1—C7—C8—Cl2	−71.0 (3)
C9—C3—C4—C10	0.5 (5)	N1—C7—C8—Cl2	108.2 (3)
C3—C4—C5—C6	−2.2 (5)		

### Hydrogen-bond geometry (Å, °)

**Table:**

*D*—H···*A*	*D*—H	H···*A*	*D*···*A*	*D*—H···*A*
N1—H1N···O1^i^	0.84 (4)	2.07 (4)	2.894 (3)	166 (3)

Symmetry codes: (i) *x*, −*y*+1/2, *z*−1/2.
